# A Bayesian Framework to Account for Complex Non-Genetic Factors in Gene Expression Levels Greatly Increases Power in eQTL Studies

**DOI:** 10.1371/journal.pcbi.1000770

**Published:** 2010-05-06

**Authors:** Oliver Stegle, Leopold Parts, Richard Durbin, John Winn

**Affiliations:** 1 Max Planck Institutes Tübingen, Tübingen, Germany; 2 University of Cambridge, Cambridge, United Kingdom; 3 Wellcome Trust Sanger Institute, Hinxton, Cambridge, United Kingdom; 4 Microsoft Research, Cambridge, United Kingdom; Broad Institute of MIT and Harvard, United States of America

## Abstract

Gene expression measurements are influenced by a wide range of factors, such as the state of the cell, experimental conditions and variants in the sequence of regulatory regions. To understand the effect of a variable of interest, such as the genotype of a locus, it is important to account for variation that is due to confounding causes. Here, we present VBQTL, a probabilistic approach for mapping expression quantitative trait loci (eQTLs) that jointly models contributions from genotype as well as known and hidden confounding factors. VBQTL is implemented within an efficient and flexible inference framework, making it fast and tractable on large-scale problems. We compare the performance of VBQTL with alternative methods for dealing with confounding variability on eQTL mapping datasets from simulations, yeast, mouse, and human. Employing Bayesian complexity control and joint modelling is shown to result in more precise estimates of the contribution of different confounding factors resulting in additional associations to measured transcript levels compared to alternative approaches. We present a threefold larger collection of *cis* eQTLs than previously found in a whole-genome eQTL scan of an outbred human population. Altogether, 27% of the tested probes show a significant genetic association in *cis*, and we validate that the additional eQTLs are likely to be real by replicating them in different sets of individuals. Our method is the next step in the analysis of high-dimensional phenotype data, and its application has revealed insights into genetic regulation of gene expression by demonstrating more abundant *cis*-acting eQTLs in human than previously shown. Our software is freely available online at http://www.sanger.ac.uk/resources/software/peer/.

## Author Summary

Gene expression is a complex phenotype. The measured expression level in an experiment can be affected by a wide range of factors—state of the cell, experimental conditions, variants in the sequence of regulatory regions, and others. To understand genotype-to-phenotype relationships, we need to be able to distinguish the variation that is due to the genetic state from all the confounding causes. We present VBQTL, a probabilistic method for dissecting gene expression variation by jointly modelling the underlying global causes of variability and the genetic effect. Our method is implemented in a flexible framework that allows for quick model adaptation and comparison with alternative models. The probabilistic approach yields more accurate estimates of the contributions from different sources of variation. Applying VBQTL, we find that common genetic variation controlling gene expression levels in human is more abundant than previously shown, which has implications for a wide range of studies relating genotype to phenotype.

## Introduction

DNA microarray technologies allow for quantification of expression levels of thousands of loci in the genome. These measurements enable exploring how a variable, such as clinical phenotype, tissue type, or genetic background, affects the transcriptional state of the sample. Recently, gene expression levels have been studied as quantitative genetic traits, investigating the effect of genotype as the primary variable. Studies have found and characterised large numbers of expression quantitative trait loci (eQTLs) [1]–[3], exploring their complexity [2], population genetics [4], [5] and associations with disease [6], [7].

**Figure 1:**
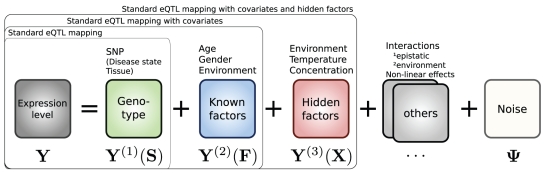
General additive model for sources of gene expression variability. The 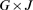 matrix 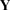 of measured gene expression levels of 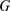 genes from 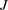 individuals is modelled by additive contributions from components 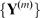 and observation noise 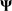. Here, the components capture the signal due to primary effect of the genetic state 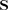, known factors 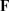 and hidden factors 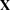. Some examples of possible underlying sources of variation are given above the model boxes. The groupings represent some standard genetic association models commonly used.

An important issue in such studies is additional variation in expression data that is not due to the genetic state, as illustrated in Figure 1. Intracellular fluctuations, environmental conditions, and experimental procedures are factors that all can have a strong effect on the measured transcript levels [2], [8]–[10] and thereby obscure the association signal. When measured, correct estimation of the additional variation due to these *known factors* allows for a more sensitive analysis of the genetic effect. For example, it has been reported that additional human eQTLs can be found when including the known factors of age, and blood cell counts in the model [7]. It is also standard procedure to correct for batch effects, such as image artefacts or sample preparation differences [11].

In practise it is not possible to measure or even be aware of all potential sources of variation, but nevertheless it is important to account for them. Unobserved, *hidden factors*, such as cell culture conditions [12] often have an influence on large numbers of genes. We and others have proposed methods to detect and correct for such effects [9], [13], [14]. These studies demonstrated the importance of accounting for hidden factors, yielding a stronger statistical discrimination signal.

The challenge in modelling several confounding sources of variation (Figure 1) is to correctly estimate the contribution that is due to each one of them. There are open questions how to ensure that only spurious signal is eliminated by methods that account for hidden factors (see for instance discussion in [14]), and how to deal with situations when both known and hidden factors are present. The problem of identifying the correct causes of the signal is even harder in the presence of additional sources of variability. For example, when searching for epistatic or genotype-environment interactions, the primary effects of other known factors and hidden factors also need to be accounted for.

The key for correctly attributing expression variability is controlling the complexity of the statistical models for each source of variation. For example, the number of genotypes considered in an association scan can be enormous, and not all of them affect the expression level of every probe. Threshold values, obtained from likelihood ratio statistics or empirical p-value distributions, can be used to determine the significance of individual associations, thereby avoiding overfitting by controlling the model complexity [4], [15]. Similar measures are necessary for models of other sources of variability such as hidden factors.

In this work we present VBQTL (Variational Bayesian QTL mapper), a joint Bayesian framework for gene expression variability that accounts for the signal from genotype, known factors, and hidden factors. VBQTL is implemented within a general framework that provides commonly used models for sources of phenotypic variation, which can be combined as needed. While previous attempts have been specific to a narrow set of underlying sources, our approach is flexible and can be adapted to a particular study design. The probabilistic treatment allows uncertainty to be propagated between models, and yields a posterior distribution over model parameters. Complexity control is tackled at the level of individual models, where parameters are regularised in a Bayesian manner.

We compare the performance of VBQTL with existing approaches for detecting expression QTLs. A simulation experiment contrasts VBQTL with common approaches that use non-Bayesian techniques for distinguishing global hidden factor effects from genetic effects. This study highlights differences in the methodology to control model complexity with implications to eQTL detection power. The necessity and difficulty to account for variability that confounds the genetic signal is demonstrated. Results on datasets from a human outbred population and crosses of inbred yeast and mouse strains show that VBQTL identifies more significant associations than alternative methods. Finally, we apply VBQTL to perform a whole-genome eQTL scan on the HapMap phase 2 expression and genotype data, demonstrating the scalability of our framework to large numbers of samples and probes. We find three times more *cis* eQTLs than a standard association mapping method, suggesting more extensive genetic control of gene expression by common variants than previously shown.

## Methods

**Figure 2:**
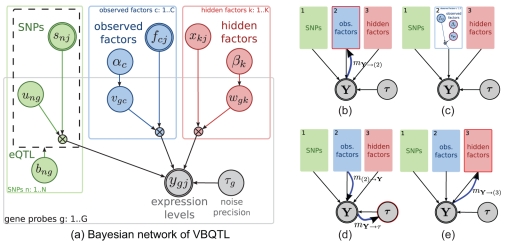
Bayesian network and outline of the inference schedule for VBQTL. (**a**) The Bayesian network for the model of gene expression variation used in VBQTL (see Methods). The full model combines genetic (green), known factor (blue) and hidden factor (red) models to explain the observed gene expression levels 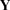. The solid rectangles indicate that contained variables are duplicated for each gene probe (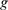), SNP (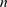) or factor (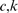) respectively. A similar rectangle for individuals (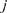) is omitted in this representation. The dashed rectangle indicates that the variable 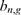 switches the contained part of the graph on or off representing the existence or lack of an association. Nodes with thick outlines (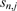, 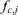 and 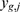) are observed. (**b**)–(**e**) Update cycle of the known factors model introduced in Section Inference. The red outline highlights the parts of the model that change in a step, and the thick blue arrows illustrate the flow of information. Details of these updates are discussed in the text.

Here, we present VBQTL, a configuration of a general framework for modelling diverse sources of gene expression variability. The model underlying this framework assumes that gene expression levels are influenced by additive effects from independent sources, e.g. in the case of VBQTL these are contributions from genotype, known factors, and hidden factors (Figures 1, 2a). We cast the full model in a probabilistic setting, treating its parameters as random variables.

We perform Bayesian inference in the joint model, which is appealing for several reasons. First, it allows possible dependencies between the different sources of variation to be captured. The effects of the genotype, known and hidden factors are learned jointly, taking other parts of the model into account. Propagation of uncertainty leads to more accurate parameter estimates [16], and avoids possible pathologies, for instance of maximum likelihood methods [17]. Second, Bayesian inference allows different models to be flexibly combined according to the needs of a particular study. Many existing approaches can be cast as special cases of this general framework, with some examples given in Figure 1. Finally, the Bayesian approach leads itself to efficient approximate inference schemes such as variational methods [18], rendering the resulting algorithms applicable to large-scale and high-dimensional datasets. Also, variational learning allows an inference schedule to be specified by the user, leading to distinct algorithms with different computational complexity and properties (see Inference).

In the following, we present the mathematical model of VBQTL, and an outline of the inference procedure. We then describe alternative non-Bayesian models for expression QTL studies used in the experiments. An in-depth treatment of the framework including full details about the parameter estimation is provided in Text S1.

### VBQTL - a joint Bayesian model for gene expression variability

The observed gene expression matrix 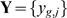 for genes 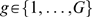 and individuals 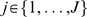 is modelled by the sum of contributions 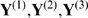 from the genotype, known and hidden factor models and Gaussian noise with precisions 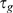 for each gene 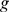


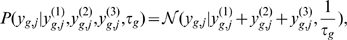
(1)with a gamma prior on the noise precisions 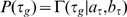 (Figure 2a). The 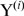 comprise the contribution of individual sources to the variability in the observed expression levels, and are themselves treated as random variables with different underlying models.

**1) Genotype effect model** represents the probabilistic variant of the standard genetic association model, where some of the SNP genotypes have a linear effect on gene expression levels. The genetic component of the expression level 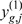 of the 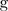
th gene probe in the 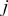
th individual is explained by linear effects of the genotypes of 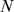 SNPs 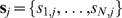 (Figure 2a, green plate):
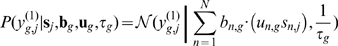
(2)

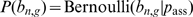
(3)

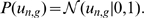
(4)The weights 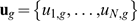 control the magnitude of the effect of the SNP on the expression levels of genes 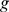. The binary variables 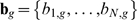 determine whether the SNP effect is significant (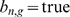) or not (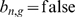). The prior probability 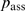 of an individual association controls the complexity of the model by influencing the a priori expected number of significant associations; this parameter corresponds to a significance threshold in a classical setting (Text S1).

To reduce the computational cost, inference in the association model is approximated, only considering a single most relevant SNP-regulator per gene, with the other 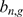 forced to 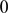. This bottleneck approximation ensures tractability of the joint association model for large-scale studies (Text S1), avoiding the need to track the covariance between effects from multiple SNPs.

**2) Known factor model** accounts for the effect of known covariates 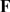 of individual samples, such as environmental conditions, gender, or a population indicator. The linear effects of 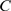 measured covariates in the 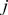
th individual, 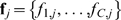, is taken into account using a variant of Bayesian regression (Figure 2a, blue plate):
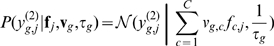
(5)

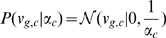
(6)

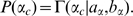
(7)Here, 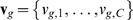 is the corresponding weight vector for each gene 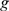. The gamma prior on the inverse variance 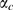 for weights of each factor introduces automatic relevance detection (ARD) [19], [20], driving the weights of unused factors to 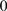 and thereby switching them off. This provides complexity control of the model by regularising the effective number of covariates.

**3) Hidden factor model** accounts for the effect of hidden factors (such as unmeasured covariates and global effects) on the gene expression levels. We use a probabilistic variant of the classical factor analysis model for this task. We have previously shown that this model captures hidden factors better than alternative linear models, such as probabilistic principal component analysis or independent component analysis [13]. Similarly to known factors, the expression level of gene 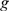 in individual 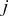 is modelled by linear effects from a chosen number of 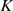 hidden factors 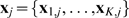 (Figure 2a, red plate).
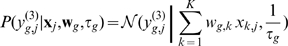
(8)

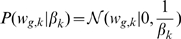
(9)

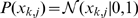
(10)

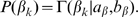
(11)Note that in contrast to the known factor model, the factor activations 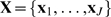 are unobserved random variables that need to be inferred from the expression profiles. Again, the ARD prior switches unused factors off, thereby providing probabilistic complexity control ([13], Results).

### Inference

Parameter inference in VBQTL is implemented using variational Bayesian learning [18], a generalisation of the expectation maximisation algorithm. An approximate 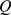-distribution over model parameters is iteratively refined until convergence. In each iteration, approximate distributions of individual parameters are updated according to a specified schedule, taking the current state of all other parameter distributions into account (Figure 2b–e). Choosing an approximation that factorises over individual models, the variational update equations have an intuitive interpretation:

1. The current belief of the residual dataset for a particular active model is calculated, taking the prediction form all other models and the estimated noise precision into account (Figure 2b).
2. The parameters of the active 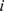
th model are updated based on their previous states and the new residual dataset (Figure 2c).
3. The distribution of the model contribution 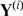 is recalculated using the updated parameter values. The global noise precisions 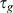 are updated (Figure 2d) based on the first and second moments of all the contributions.
4. The same procedure is in turn applied to the remaining models in the schedule (Figure 2e) until convergence.

This iterative procedure, performing updates of local parameter distributions in turn, can be interpreted as a message passing algorithm, where sufficient statistics of parameter and data distributions are propagated across the graphical model [21].

The initial values of parameters are determined from maximum likelihood solutions. A random initialisation via sampling from the prior is possible as well; we have not explored the implications of this alternative here. Details on inference and the individual parameter update equations are given in Text S1.

In experiments, we compare two alternative inference schedules of VBQTL. In iterative VBQTL (iVBQTL), the model parameters are learned using several iterations through all model components, first updating the genetic model, then known and hidden factors (Text S1). An important property of iVBQTL is that hidden factors are estimated jointly with the genetic state and known factors. This choice of schedule and the iterative learning help to ensure that variability that is due to genetic associations is not explained away by other parts of the model (Results).

In cases where neither known nor hidden factors are correlated with the genetic state, their effect can be learned independently without running the risk of explaining away meaningful association signal. This motivates fast VBQTL (fVBQTL), which performs a single update iteration of the full model, first inferring the contribution from the known and hidden factors, and then from the genetic state. This simpler schedule can save significant computation time, since the factor effects can be precalculated, and only a single iteration of the computationally more expensive genetic association model is needed. In cases where the genetic state is approximately orthogonal to the known and hidden factors, this cheaper approximation performs equally with iVBQTL for finding genetic associations (Results).

### Alternative methods to account for confounding variation in expression QTL studies

We compared VBQTL with previous methods that account for confounding variance in the context of expression QTL mapping. Similarly to VBQTL, they model known and hidden factors in the expression levels. The differences between the alternative methods are in the hidden factor model used, which in turn vary in the complexity control approach employed as highlighted below. Thus these alternative models are named after the hidden factor estimation method.

- **Standard model** explains the expression variability solely by the effects of known factors and SNP genotypes, without accounting for the hidden factors.
- **PCA** uses principal component analysis to detect hidden factors. In general, PCA can explain all the variability in the data. Complexity is controlled by specifying the number of components to use as a parameter.
- **PCAsig** is an extension of PCA to account for hidden factors. In this model, complexity control is achieved via significance testing of eigenvalues, retaining only components that explain more variance than expected by chance at a specified significance cutoff (Text S1).
- **SVA** model controls complexity similarly to PCAsig, and also accommodates a per-gene noise model and explicitly allows for sparse non-orthogonal components [9].

For a quantitative evaluation of the performance of each method, we considered the resulting residuals of the estimated effects from known and hidden factors. To detect eQTLs we applied standard statistical tests employing a linear model on the SNP genotype on these residual datasets (Text S1). For iVBQTL and fVBQTL, we inferred the posterior parameter distributions, and subtracted off the estimated effect of known and hidden factors. For other methods, we first subtracted off the standard linear regression fit of the known factors, and then learned and subtracted off the hidden factor effects on the residuals. All these alternative methods are also implemented in the general framework; for details see Text S1.

While VBQTL shares basic assumptions with these alternatives, there are a number of differences. First, it is a probabilistic model that operates with uncertainties in the parameter estimates as explained above. Second, the hidden factor model allows for non-orthogonal components, and provides probabilistic complexity control based on ARD. Third, the iVBQTL schedule takes the genetic signal into account when estimating the hidden factor effect. Finally, the VBQTL model estimates a global gene-specific noise level, while the non-Bayesian models either estimate noise levels implicitly (SVA) or assume noise-free observations (PCA, PCAsig).

## Results

### Simulation study highlights performance differences due to complexity control approaches

We employed a simulated dataset to highlight the differences between alternative approaches to account for global factors in eQTL finding. Our synthetic expression data combines linear effects from genetic associations (eQTLs), known, hidden, and genetic global factors, and gene-specific noise (Text S1). We used three known and seven unknown global factors whose influence varies significantly to simulate effects with a range of magnitudes. These factors are meant to represent sources of confounding variation that are encountered in the study of the real datasets. We also introduced three global genetic factors giving rise to *trans* eQTL hotspots, mimicking the action of a genetic variant in a transcriptional regulator (e.g. transcription factor or pathway component). Such loci have been observed in several eQTL mapping studies [1], [3]. We designated three genes with a simulated eQTL as such regulators, and simulated correlated expression levels for 15% of the genes for each. While the specific simulation scenario may be biased in the comparative performance of different methods, its underlying linear model is shared by all the considered approaches, and it gives intuition for the results on real datasets discussed later.

#### Complexity control determines the accuracy of the hidden factor model

We assessed the ability of the considered methods to recover the simulated confounding variability. For those approaches that do infer hidden factor effects, we varied the corresponding complexity control parameters to investigate the influence on performance. For methods that take the number of components in the hidden factor model as a parameter (PCA, VBQTL), performance for one to 50 hidden factors was compared. For significance-testing based methods, we considered different significance cutoffs 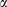 in the range 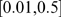.

**Figure 3:**
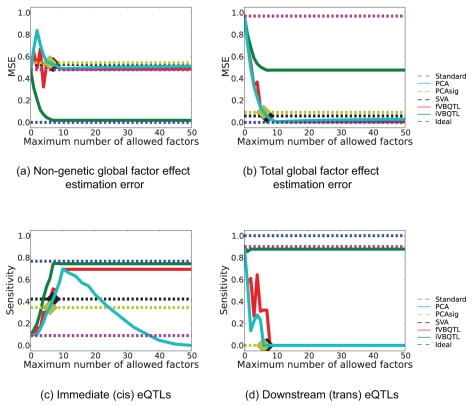
Sensitivity of recovering simulated hidden factor effects and eQTLs for Bayesian and non-Bayesian methods. (**a**) Mean-squared error in estimating only the hidden factor contribution. Methods that do not explicitly retain the genetic factors explain them away as hidden global factors, resulting in high error comparable to not accounting for hidden factors at all (Standard). (**b**) Mean-squared error in estimating the contribution from hidden and genetic factors. (**c**) Sensitivity of recovering immediate SNP associations. (**d**) Sensitivity of recovering downstream associations. Seven hidden factors and three transcription factor effects were simulated. For eQTL sensitivity, standard eQTL finding on simulated data (Standard) and same data without the hidden effects (Ideal) are included as comparisons. PCAsig and SVA identified a constant number of hidden components (marked with a diamond shape), thus only a single result (dashed line) is given.

iVBQTL correctly captured the non-genetic global factor effects (Figure 3a), as it is the only method that models the genetic signal when learning hidden factors. All other methods treat the simulated transcription factor contributions as confounding variation and explain them away. This can be a desired effect when the genetic signal is not of primary interest, or a serious shortcoming when downstream eQTLs are sought.

Complexity control settings determined the performance of capturing the simulated global effects on expression levels. PCA was most accurate when the number of hidden factors was set to 10, since seven hidden factors and three transcription factors were simulated. For larger number of components PCA overfitted, and started explaining away genetic signal, resulting in the increase in error. For a small number of components, transcription factor effects were explained away first, which increased the error in estimating the hidden factors alone. However, the estimates of the total global effects improved. PCAsig and SVA found 6 and 7 significant hidden factors for the wide range of significance cutoffs, 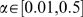, respectively. They failed to detect some of the weaker hidden effects that continued to mask the genetic signal, and underfitted the data. Their performance was similar to PCA with the matching number of components. While the significance-testing based complexity control prevents these approaches from overfitting, only a single outcome is observed for a wide range of parameter settings, with the models settling to a rigid suboptimal solution. fVBQTL achieved the most accurate estimation of global variation. Notably, unlike PCA, its performance did not degrade for large numbers of hidden factors in the model, exhibiting good complexity control in this scenario.

#### Hidden factor effect estimation accuracy is mirrored in eQTL finding sensitivity

We determined the sensitivity and specificity of the considered methods for detecting the immediate and downstream simulated genetic associations. The significance of an eQTL was tested using a two-sided t test on the correlation coefficient with a 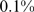 Bonferroni corrected per-gene false positive rate in the genetic association model. The results when calling eQTLs using regression on ranks, or permutations to establish the empirical null distribution of LOD scores were almost identical (Figure S1. As a benchmark, the comparison includes eQTL finding using the standard method on both raw expression data (Standard), and an ideal case, where the simulated hidden factor effects are removed, but the simulated genetic factors maintained (Ideal).

The accuracy of the hidden factor effect estimation mirrored the immediate eQTL finding sensitivity (Figure 3c). The specificity was consistent with the chosen false positive rate for all methods (data not shown). fVBQTL and iVBQTL recovered more true *cis* eQTLs compared to other methods, approaching the performance of the ideal case, mirroring the accuracy of estimating hidden factor effects. PCA overfitted when the number of components used was greater than the true number of ten simulated global factors, explaining away genetic signal. While the PCA error for detecting global effects increased only marginally, the decrease in sensitivity for identifying eQTLs was severe. The overfitting in case of PCA, and underfitting in case of PCAsig and SVA both resulted in a loss of sensitivity to find the simulated *cis* associations. fVBQTL and iVBQTL did not suffer from either deficiency, capturing nearly all the associations possible in the ideal case.

All methods except iVBQTL and standard method explained away simulated *trans* eQTL hotspots (Figure 3d). This is due to the global factor effect estimation accuracy, where iVBQTL alone refrained from explaining the hotspots away as a global factor. The standard method found nearly all the original *trans* associations, actually outperforming methods that explain away confounding variability. Thus, in cases where there is true genetic signal with widespread downstream effects, its contribution needs to be taken into account to retain its relation to genotype, and avoid attributing it to a confounding global cause. This is straightforward in our framework, and is demonstrated by the good performance of iVBQTL in this scenario. iVBQTL retained the original associations, while explaining away non-genetic causes of expression variability, thus adding power to detect the weaker, masked eQTLs. This effect is also observed in the study of crosses of inbred strains below.

Taken together these results suggest that it is important to account for the confounding sources of variation in expression levels, while keeping the signal of the genetic state. Correct complexity control is required to avoid over- and underfitting in order to achieve optimal sensitivity for detecting true genetic associations.

### VBQTL finds additional expression QTLs in real datasets

Next, we compared the same methods for expression QTL finding on yeast [2], mouse [3] and human [4] datasets. These represent common study designs of an outbred population (human), and a population of crosses between inbred strains (yeast, mouse). We considered 5, 15, 30, and 60 hidden factors for PCA and VBQTL, and 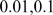, and 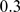 as significance cutoffs for SVA and PCAsig. Expression QTLs were detected using a two-sided t test analogously to the simulation scenario. Again, results for alternative genetic association tests were similar (Figures S2, S3, S4).

#### Accounting for hidden factors helps to detect additional *cis* eQTLs in an outbred population

We applied the considered methods on the genotype and expression data from 90 individuals of the CEU (CEPH from Utah) HapMap phase 2 samples [4], [22]. The data consisted of genotypes of 55,000 SNPs and expression levels of 618 probes from chromosome 19 (results for three more chromosomes were similar, data not shown). The expression levels were measured in EBV-transformed lymphoblastoid cell lines of healthy individuals. The gender covariate was included as a known factor for all methods. We did not consider probes with overlapping SNPs. Following [4], an association was called to be in *cis* when the SNP was within 1Mb from the probe midpoint and in *trans* otherwise.

**Figure 4:**
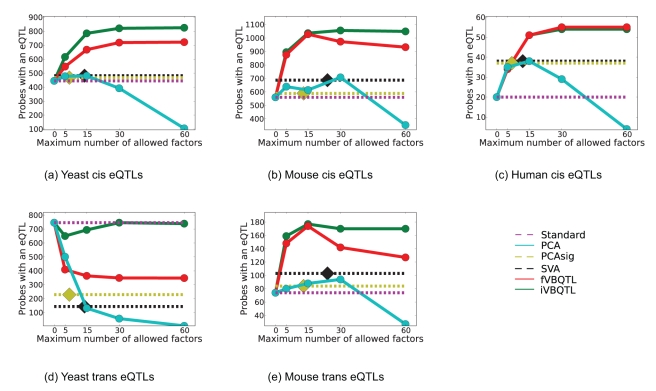
Number of probes with an eQTL found as a function of maximum number of hidden factors for three previously published datasets. Significance-testing based methods (PCAsig, SVA) identified the same number of factors for a wide range of cutoff values (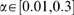), thus only a single count is given (dashed lines), together with the number of factors found (diamond shape). Other methods were applied with a maximum number of 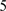, 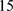, 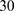 and 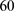 hidden factors.

The standard method found the least gene probes with a *cis* association (20, Figure 4c), suggesting that strong confounding sources of variation are present in this dataset. The number of identified probes with a *trans* association was not significantly higher than expected by chance at the chosen FPR, which is in line with previous results [4], and suggests little intrachromosomal *trans* regulation.

PCA, the simplest method for accounting for hidden factors, found additional associations when up to 30 principal components were used, but substantially fewer for 60 components. This is expected, since there are no more than 90 degrees of freedom in this dataset, and 60 principal components accounted for over 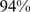 of the variance (Table S5), and hence PCA is likely to explain away part of the genetic association signal for large numbers of components.

The significance-testing based methods, SVA and PCAsig both found additional associations compared to the standard method. It is remarkable that both found a constant number of significant hidden factors for the wide range 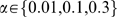 of significance cutoffs considered, again exhibiting rigid complexity control. The performance of SVA with the 12 hidden factors found is close to performance of PCA with 15 components (both find 38 probes with an association). Similarly, PCAsig with the 7 significant components performs comparably to PCA with 5 components (37 vs. 35 probes with an association). This shows the intrinsic similarity of these methods to PCA, as was also observed in the simulation scenario.

fVBQTL and iVBQTL found more probes with an association (55 and 54) than all other methods, representing an almost threefold increase in the number of genes with a *cis* eQTL. Complexity control assured that the performance saturated for large enough number of factors and did not degrade as for PCA. None of the estimated hidden factors was significantly correlated to a SNP genotype, suggesting that individual genetic variants do not have global effects on many gene expression levels in this dataset.

It is important to note that the model performance depends on two aspects. First, the model complexity control, regulating the amount of variance explained, is important to ensure that genetic signal is not attributed to hidden factors. Overfitting in case of PCA for a large number of components is an example of such an effect. Second, while alternative hidden factor models explained similar amounts of variance, their performance differed due to the underlying model. For example, PCA and fVBQTL both explained about 70% of variance in the observed expression levels (Table S5), yet fVBQTL identified additional associations. These findings are consistent with the simulation study results, and suggest that the additional associations found with Bayesian models are due to differences in the underlying model and complexity control.

#### Accounting for hidden factors adds power to detect *cis* associations in crosses between inbred mouse and yeast strains

Next, we applied the methods to two datasets of inbred strain crosses. The yeast expression dataset [2] (GEO [23] accession GSE1990 with genotypes provided by authors) contained 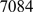 expression measurements and 2925 genotyped loci in 112 crosses of segregating yeast strains. The mouse expression data [3] consisted of 23,698 expression measurements for 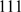 F_2_ mouse lines, and genotypes at 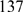 genetic markers. An association was called to be in *cis* if the probe and the genotyped locus were from the same chromosome, and in *trans* otherwise.

The relative performance of different methods was similar to their ability to detect *cis* eQTLs in the outbred population dataset (Figures 4a, 4b). The absolute performance gain was significantly lower for all methods, however. This finding suggests that the genetic signal is stronger compared to confounding sources of variation, which is not unexpected from the study design. All factor methods identified additional associations compared to the standard method. PCA overfitted for larger numbers of principal components used, explaining away genetic association signal. SVA and PCAsig found the same number of significant hidden factors for a range of significance cutoffs considered, exhibiting little flexibility. Again, their performance was similar to extrapolation of PCA results with matching numbers of effective components. fVBQTL and iVBQTL found additional genetic associations in *cis* compared to the standard model and other methods for accounting for confounding variance, as observed in simulations and human dataset. Summary statistics for the method performance can be found in Table S6 and S7 respectively.

#### Iterative learning with iVBQTL overcomes difficulties in detecting *trans* associations for crosses of inbred strains

All methods found additional *trans* associations in mouse, but fewer than the standard method in yeast (Figure 4d, 4e). In yeast, the more variance was explained by the hidden factors, the fewer *trans* eQTLs were found, suggesting that the global determinants of gene expression variation were correlated with the genetic state. Indeed, the inferred hidden factor levels were correlated with genotypes of “pivotal loci” that are associated with expression levels of hundreds of genes.

The effect of pivotal loci has been observed before, and interpreted in different ways [9], [14]. It could be that the additional variation is artefactual, and correlated to the genetic state by chance. In this case, all the original *trans* associations are spurious. The alternative explanation is that the genotype of these loci have real downstream effects on the expression profiles of the genes. In this case the variance is not confounding the genetic signal, but in fact is a part of it, and hence should not be explained away.

Previous methods do not provide consistent ways of dealing with this issue. The SVA authors also suggest to remove the effect of the primary variable first. However, the authors do not consider accounting for the genetic effect in their application to the same yeast dataset [9]. In a second study [14], the application of a correction procedure also explains away *trans* associations. We provide a principled approach for dealing with this situation and show its merit. The iVBQTL scheduling takes the genetic state into account while learning the hidden factors, and as a consequence is more sensitive to genetic associations.

### Application of VBQTL recovers three times more probes with a *cis* eQTL in a whole-genome scan of HapMap phase II data

Motivated by the results of the initial study of a single human chromosome, we applied fVBQTL, learning 30 hidden factors, to the 10,000 most variable expression probes of the HapMap 2 dataset. We searched for *cis* eQTLs in the original expression data (standard eQTLs) as well as the residuals of fVBQTL (VBeQTLs), using a 2-tailed t test with 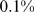 Bonferroni-corrected per-gene FPR to assess the significance of association.

**Figure 5:**
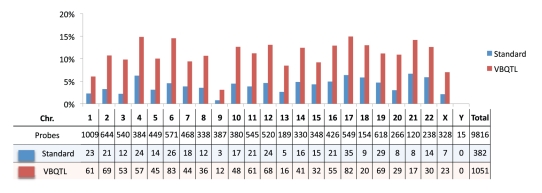
Fraction of tested genes with a *cis* association in individual chromosomes and overall false discovery rate for the HapMap CEU population (FPR = 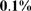).

On the CEU population, we found 1051 genes with a VBeQTL at false discovery rate (FDR) of 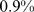, and 382 genes with a standard eQTL at FDR of 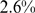 (Figure 5). This result corresponds to nearly a threefold increase in the number of genes with an association, and is consistent across chromosomes. A similar increase in the number of associations was found for other populations (Table S1).

**Figure 6:**
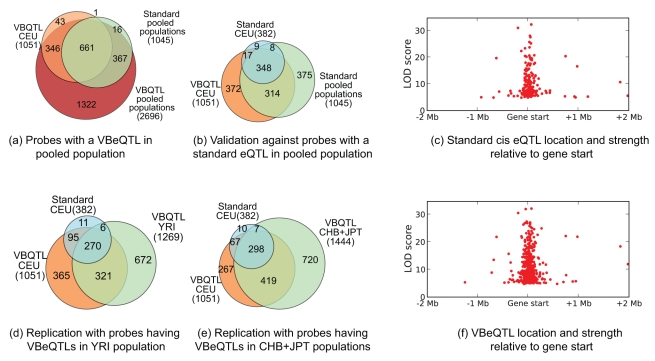
Validation of VBeQTLs by comparison to standard eQTLs. (**a,b,d,e**) Venn diagrams depicting overlap of probes with a standard eQTL or VBeQTL in the CEU population and probes with an eQTL in other populations. (**c,f**) Standard and VBeQTL location and strength relative to the transcription start site.

We repeated this genome-wide experiment on pooled populations. Due to the increased sample size, it was possible to detect additional associations. We found 2696 genes with a VBeQTL compared to 1045 genes with a standard eQTL at the 0.1% FPR (Figure 6a). The VBeQTLs in the pooled sample cover 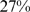 of all the considered probes, suggesting that the number of human genes whose expression levels are affected by common *cis*-acting genetic variation may be significantly higher than previously shown [24], [25]. This additional abundance of associations suggests that detection of *cis* eQTLs has not been saturated and larger sample sizes may lead to evidence of even more extensive *cis* regulation by common polymorphisms.

Exploratory results indicate additional power to find *trans* eQTLs without explaining away eQTL hotspots (Text S2). These should be interpreted with caution due to very stringent multiple testing corrections, however.

### Additional associations are due to increased sensitivity

It is important to demonstrate that the additional associations found after removing the learned non-genetic factors are biologically meaningful. We provide evidence that the additional associations found in HapMap phase 2 data are real in three ways.

First, we investigated how many of the genes with a VBeQTL in each of the three populations individually were replicated using the standard method on a pooled data set containing all populations. Note that this will only validate weak associations that occur in multiple populations – we would not expect weak population-specific associations to be replicated in the pooled data set. However, we expect many of the associations to be replicated in multiple populations [24]. A total of 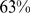 of all and 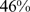 of the additional associations found in the CEU population were recovered using the standard method in the pooled population (Figure 6b). The remaining additional associations may be explained by even weaker signals that were recovered by applying fVBQTL, or as population-specific effects that do not stand out in the pooled sample. Analogous overlaps were found when excluding the CEU population from the pooled analysis (Table S3).

Second, we evaluated to what extent the additional genes with a VBeQTL in a single population were replicated in other populations. For instance, 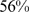 of genes with a CEU VBeQTL were replicated on the YRI population (Figure 6d), and 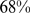 on the CHB+JPT population (Figure 6e). These overlaps are consistent with overlaps of standard eQTLs, and are similar for other populations (Table S2), and alternative methods accounting for hidden factors.

Finally, we validated that the locations of the novel associations are distributed similarly to the original ones. We analysed the distribution of the position of additional *cis* associations around the gene start along with the association LOD scores. The additional VBeQTLs have very similar characteristics to the standard eQTLs, being concentrated around the gene start (Figure 6c, 6f), in line with previous results [24].

### Interpretation of learned hidden factors

The hidden factor models hypothesise a set of unobserved non-genetic factors that influence the measured gene expression levels. To gain insights into their interpretation we considered correlations to known effects such as gender, population or environment, and the sets of genes most influenced.

We applied fVBQTL to expression data from individuals of all three HapMap populations, and tested for correlation between the inferred hidden factors and the population and gender indicator variables. The resulting correlation coefficients (Table S4) indicate that many of the learned latent causes are correlated with population and that one is strongly correlated with gender. This implies that the hidden factor model can recapture variance in the gene expression levels due to true underlying properties of individuals. However, none of the global factors learned in one population was correlated with a single SNP genotype.

A recent study in yeast looked for changes in eQTLs when segregating strains were grown in different media [26]. We applied fVBQTL to the expression data of this study (GEO accession GSE9376), without including any information about the growth condition. The first hidden factor learned was highly correlated with the indicator variable for the growth condition (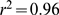), demonstrating that the VBQTL model can successfully recover an environmental effect if it is present.

The global factors identified can be further analysed for biological signals, looking for GO term over-representation in the genes that they affect. We used the ordered GO profiling method [27] to find significantly enriched GO categories for 30 genes most affected by each factor. Recent results [28] show that related linear Gaussian models find biologically relevant factors in the yeast expression dataset. We replicated these findings with our model, yielding factors enriched in biological functions, including sugar, alcohol and amino acid metabolic processes. Similar analysis in human and mouse did not show significant over-representation of GO categories, providing no evidence that the main axes of variation in the expression levels for these experiments are due to common biological function. This could be due to poor annotation of the genes, gene features not related to biological function, or more technical sources of global variation, such as cell culture conditions [12].

## Discussion

We have presented VBQTL, a probabilistic model to dissect gene expression variation in the context of genetic association studies. The model is implemented in a Bayesian inference framework that allows uncertainty to be propagated between different parts of the model, and yields posterior distributions over parameter estimates for more sensitive analysis. In comparative eQTL mapping experiments, VBQTL outperformed alternative methods for eQTL finding on simulated and real data. In the most striking example, VBQTL found up to three times more eQTLs than a standard method, and 45% more compared to the best alternative in the HapMap 2 expression dataset.

Our approach advances the methodology for understanding phenotypic variation. The implementation of a flexible framework allows models for explaining the observed variability to be straightforwardly combined. Notably, non-Bayesian models can also be included, as we demonstrated with PCA, SVA, and linear regression models. VBQTL controls the model complexity at the level of all individual components of expression variability, thereby preventing from over- and underfitting. Our experimental results on simulation and real data showed how explaining away too much variability removes some signal of interest from the data, and failing to account for all sources of confounding variation decreases power to detect the relevant signal. When the variable of interest is correlated with many gene expression levels, its effect can be falsely explained away by the hidden factor model. We showed that in such settings the choice of an iterative schedule helps to ensure that variability is explained by the appropriate part of the model. There can be no silver bullet solution that provides perfect results in any scenario with no supervision. Instead, modelling assumptions must be made explicit, and incorporated in the analysis, as is elegantly done in the Bayesian setting.

VBQTL and other methods that account for hidden factors all found additional expression QTLs in the datasets studied compared to the standard method. It is remarkable that, with only 270 samples, and looking in one tissue type, we can find significant genetic associations to 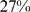 of the expressed genes. While similar results have been reported before, we have shown a threefold increase in the number of associations for the HapMap dataset, and analysed their repeatability and location distribution. The replication of the additional associations in different populations suggests that they are genuine. The increase in power is due to the hidden factor model, which explains away unwanted non-genetic variability, thereby allowing the genetic effects to stand out to a greater extent. The high number of additional associations suggests that association finding studies in human have not saturated, and we expect the fraction of genes with an eQTL will increase further as the number of samples grows. It may be that the expression of majority of human genes varies as a result of segregating genetic variation. While previous studies have reported only 12% of heritable variation to be due to *cis* variants [29], this does not contradict the presence of weak *cis* eQTLs for a large fraction of the genes.

In conclusion, we believe that VBQTL provides a principled and accurate way to study gene expression and other high-dimensional data. Increasingly complex models combining genetic and other effects can explain significantly more of the variance in observed phenotypes, as suggested by this study and others. Our general framework provides the flexibility to facilitate these richer models, for example, we have already started exploring interaction effects as an additional model of the framework. It will be interesting to see how these approaches can contribute to our understanding of human disease genetics, potentially involving intermediate phenotypes such as gene expression and other factors.

The software used in this study is freely available online at http://www.sanger.ac.uk/resources/software/peer/.

## Supporting Information

Text S1 Supplementary methods.
(0.23 MB PDF)

Text S2 Supplementary results.
(0.86 MB PDF)

Figure S1 Sensitivity of recovering simulated eQTLs for alternative eQTL models. (a–b) Using a standard model for expression values, performing 2-tailed t tests on the statistic based on correlation coefficient between expression level and genotype. (c–d) Similar test for ranks of expression values. (e–f) Permutation test with 1000 permutations and 0.1% FPR. Bonferroni correction to 0.1% false positive rate was used for (a–d) to correct for multiple testing as detailed in Text S1.
(0.30 MB PDF)

Figure S2 Sensitivity of recovering human eQTLs for alternative eQTL models. (a–b) Using a standard nested model for expression values, performing chi-squared tests with one degree of freedom on the log likelihood ratio for adding the genetic association term to the model. (c–d) Using a standard nested model for ranks of expression values, performing t tests with N-2 degrees of freedom as described in Supplementary Methods. Bonferroni correction to 1% false positive rate was used for both methods to correct for multiple testing as detailed in Text S1.
(0.23 MB PDF)

Figure S3 Sensitivity of recovering yeast eQTLs for alternative eQTL models. (a–b) Using a standard model for expression values, performing 2-tailed t tests on the statistic based on correlation coefficient between expression level and genotype. (c–d) Similar test for ranks of expression values. Bonferroni correction to 0.1% false positive rate was used for both methods to correct for multiple testing as detailed in Text S1.
(0.26 MB PDF)

Figure S4 Sensitivity of recovering mouse eQTLs for alternative eQTL models. (a–b) Using a standard model for expression values, performing 2-tailed t tests on the statistic based on correlation coefficient between expression level and genotype. (c–d) Similar test for ranks of expression values. Bonferroni correction to 0.1% false positive rate was used for both methods to correct for multiple testing as detailed in Text S1.
(0.25 MB PDF)

Table S1 Number of probes with a *cis* association for individual chromosomes and per-probe false discovery rate for the considered populations (per-probe FPR = 0.100%, Bonferroni corrected for testing multiple SNPs per probe, 2-tailed t test) on raw expression data (Standard) and after accounting for hidden factors (fVBQTL).
(0.02 MB PDF)

Table S2 Magnitude and fraction of overlap between probes with a Standard of fVBQTL *cis* eQTL respectively, for different populations and methods. Total numbers for each population and method are given in parenthesis after the population. 955 probes had a standard eQTL in some population, and 148 in every population. 2236 probes had a fVBQTL eQTL in some population, and 477 in every population.
(0.02 MB PDF)

Table S3 Overlap of VBQTLs in one population (2.) with standard eQTLs found when pooling the other two populations (3.). Overlaps are given both for all QTLs (2. & 3.) and only for additional ones (2. - 1. & 3. - 1.) compared to standard eQTLs in the population. Per-probe eQTL FPR = 0.1%, Bonferroni corrected for testing multiple SNPs per probe, 2-tailed t test.
(0.01 MB PDF)

Table S4 Pearson correlation coefficient between top 6 factors learned on the pooled HapMap data, and 4 indicator variables relating to the background of the individual. Correlations with absolute value above 0.6 are highlighted.
(0.01 MB PDF)

Table S5 Summary statistics for method performances on the human chromosome 19 dataset presented in the main text. The parameters for different methods are varied by the number of allowed factors K (PCA, VBQTL) or by the significance cutoff α (PCAsig, SVA). Hidden factor summary is given by the number of factors found and the variance explained by the hidden factor effects. The number of probes with a *cis* and *trans* eQTL, as well as the sensitivity and specificity of recovering probes with a standard eQTL are given. Per-probe eQTL FPR = 0.001, Bonferroni corrected for testing multiple SNPs per probe, 2-tailed t test.
(0.02 MB PDF)

Table S6 Summary statistics for method performances on the yeast dataset presented in the main text. The parameters for different methods are varied by the number of allowed factors K (PCA, VBQTL) or by the significance cutoff α (PCAsig, SVA). Hidden factor summary is given by the number of factors found and the variance explained by the hidden factor effects. The number of probes with a *cis* and *trans* eQTL, as well as the sensitivity and specificity of recovering probes with a standard eQTL are given. Per-probe eQTL FPR = 0.001, Bonferroni corrected for testing multiple SNPs per probe, 2-tailed t test.
(0.02 MB PDF)

Table S7 Summary statistics for method performances on the mouse dataset presented in the main text. The parameters for different methods are varied by the number of allowed factors K (PCA, VBQTL) or by the significance cutoff α (PCAsig, SVA). Hidden factor summary is given by the number of factors found and the variance explained by the hidden factor effects. The number of probes with a *cis* and *trans* eQTL, as well as the sensitivity and specificity of recovering probes with a standard eQTL are given. Per-probe eQTL FPR = 0.001, Bonferroni corrected for testing multiple SNPs per probe, 2-tailed t test.
(0.02 MB PDF)
