# Supplementary material for: A Bayesian Framework to Account for Complex Non-Genetic Factors in Gene Expression Levels Greatly Increases Power in eQTL Studies
Source: PLoS Comput Biol. 2010 May 6;6(5):e1000770. doi: 10.1371/journal.pcbi.1000770 (PMC2865505; doi:10.1371/journal.pcbi.1000770)
Supplement: Text S1 — Supplementary methods. (0.23 MB PDF) [file pcbi.1000770.s001.pdf]

Supplementary Methods

Implementation of non-Bayesian models

Standard expression QTL model

To ensure a common ground when comparing different methods, we used a well established linear regression approach [1] to detect associations. For each tested SNP n with genotype $s_{n,j}$ and gene g with expression level $y_{g,j}$, we evaluated the log-odds (LOD) score

$$L_{n,g} = \log \left\{ \prod_j \frac{P(y_{g,j} \,|\, s_{n,j}, u_{n,g})}{P(y_{g,j} \,|\, \boldsymbol{\theta}_{\mathrm{bck}})} \right\}, \tag{1}$$

which assess how well a particular gene expression level is modelled when the genetic state is taken into account, compared to how well it is model-led by a background model ignoring the genetic effect. The terms $u_{n,g}$, $\boldsymbol{\theta}_{\mathrm{bck}}$ are parameters for probe g and SNP n of the genetic and background models respectively. The probe expression levels $y_{g,j}$ can either be the raw measurements, residuals after subtracting the estimated effect of hidden and known factors, or ranks for a non-parametric statistic.

Significance of an association was evaluated in three different ways:

1. **2-tailed t test on expression values** uses the Student's t distribution with $N-2$ degrees of freedom to assess the significance of the statistic $t = (N-2)^{0.5}\rho(1-\rho^2)^{-0.5}$ based on the correlation coefficient $\rho^2 = 1 - \exp(-2L_{n,g}N^{-1})$ between the genotype and the expression levels. We called an association significant if $|t|$ was greater than the $\frac{10^{-3}}{2S}$ tail of the t_{N-2} distribution, which corresponds to a 10^{-3} Bonferroni-corrected per-gene false positive rate when performing tests for S SNPs.

2. **Rank correlation** uses the same test, but on the ranks of expression values.

3. **Permutation testing** [2] repeats the analysis in Equation (1) with permuted expression levels with respect to the genetic state, calculating the distribution of null log-odds scores. An eQTL was called significant if $L_{n,g}$ was greater than $\hat{L}_{n,g}$, the δ tail of the null distribution for a given false positive rate (FPR) δ. The same set of permutations was used for all methods. To account for multiple testing, we estimated a single significance threshold $\hat{L}_g$ per gene for all tested SNPs. This was done by taking the maximum LOD score over SNPs for a given permutation and using this score distribution when estimating the δ tail [3].

The posterior of the switch variable for the probabilistic genetic model is not used for the final tests to put all methods on equal footing.

PCA

PCA can be interpreted as decomposition of the gene expression matrix $Y = (\mathbf{y}_1 ... \mathbf{y}_G)$ into a product UDV^T, where U is the matrix of left singular vectors, D is a diagonal matrix of singular values $\lambda_1 \geq \lambda_2 \geq ... \geq \lambda_N \geq 0$, and V is the matrix of right singular vectors. To apply PCA, we used U as the weight matrix W, and DV^T as the latent factors X. For the benchmark figures, illustrating the effect for different numbers of factors, we limited the number of learned factors to a given number K by setting $D_{i,i} = 0$ for $i > K$.

PCAsig

PCAsig model is an extension of PCA, where complexity is controlled by retaining only components that explain more variance than expected by chance. Significance testing of PCA components in the PCAsig

model was performed analogously to SVA [4], but without enforcing uniformity of the p-values. We found the variance explained by each component i by calculating the statistic $d_i = \frac{\lambda_i^2}{\sum_{j=1}^{N} \lambda_j^2}$. We then permuted the columns of Y L times, calculating null statistics $d_{i1}, d_{i2}, ..., d_{iL}$ analogously. Given a cutoff value α, component i was deemed to be significant if the fraction of null statistics greater than d_i was less than α.

SVA

SVA package was downloaded from http://www.genomine.org/sva, and applied to datasets with default parameters, using 100 permutations and varying only the significance cutoff. The model implementation uses a Python to R bridge provided by RPy (http://rpy.sourceforge.net), allowing to call the original code provided by the authors.

PEER framework

VBQTL and the alternative compared methods are implemented within the PEER (Probabilistic Estimation of Expression Residuals) framework. Here, we give a full self-contained treatment of the framework and the implemented inference algorithms.

Likelihood models

The likelihood model of PEER for observed expression levels $\mathbf{Y}$ is

$$P(\mathbf{Y} \mid \mathbf{Y}^{(1)}, \ldots, \mathbf{Y}^{(M)}, \boldsymbol{\tau}) = \mathcal{N}(\mathbf{Y} \mid \mathbf{Y}^{(1)} + \cdots + \mathbf{Y}^{(M)}, \boldsymbol{\Sigma}), \quad (2)$$

where $\boldsymbol{\Sigma} = \text{diag}\{\frac{1}{\tau_g}\}$ is the diagonal matrix constructed from noise precisions $\{\tau_g\}$ and $\{\mathbf{Y}^{(m)}\}$ are the contributions of expression variability for each of M models. The noise model is per gene, similar to a factor analysis model, where gamma priors are put on the noise precisions, $P(\tau_g) = \Gamma(\tau_g \mid a_\tau, b_\tau)$. In experiments we used vague gamma prior parameters, $a_\tau = 1, b_\tau = 100$. Each of the M models itself depends on parameters $\boldsymbol{\theta}^{(m)}$ and possibly other data $\mathcal{D}^{(m)}$

$$P(\mathbf{Y}^{(m)} \mid \boldsymbol{\theta}^{(m)}, \mathcal{D}^{(m)}). \quad (3)$$

Genotype effect model. The expression level $y_{g,j}^{(1)}$ of the gth gene probe in the jth individual is explained by linear effects of genotypes of N SNPs $\mathbf{s}_j = (s_{1,j}, \ldots, s_{N,j})$:

$$P(y_{g,j}^{(1)} \mid \mathbf{s}_j, \mathbf{b}_g, \mathbf{u}_g, \tau_g) = \mathcal{N}(y_{g,j}^{(1)} \mid \sum_{n=1}^{N} b_{n,g} \cdot (u_{n,g} s_{n,j}), \frac{1}{\tau_g}) \quad (4)$$

$$P(b_{n,g}) = \text{Bernoulli}(b_{n,g} \mid p_{\text{ass}}) \quad (5)$$

$$P(u_{n,g}) = \mathcal{N}(u_{n,g} \mid 0, 1). \quad (6)$$

The weight $\mathbf{u}_g = (u_{1,g}, \ldots, u_{N,g})$ indicates the magnitude of the effect, and the binary variables $\mathbf{b}_g = (b_{1,g}, \ldots, b_{N,g})$ determine whether it is significant (true) or not (false), taking the Bernoulli prior on the switch variable $P(b_{n,g}) = \text{Bernoulli}(b_{n,g} \mid p_{\text{ass}})$ into account. When the switch variable is on, the expression level is linearly influenced by the SNP, and unaffected otherwise. The LOD score of the association model (Section Standard expression QTL model) is closely related to the switch variable $b_{n,g}$. For a particular parameter setting, the posterior probability over the switch state $b_{n,g}$ is a monotonically increasing function of the LOD score. The exact relation is $P(b_{n,g} \mid y_{g,j}, s_{j,n}) = \sigma(\text{LOD score})$ where $\sigma()$ is the sigmoid function $\sigma(x) = 1/(1 + e^{-x})$.

2) Known factor model. The effect of the measured C covariates in the jth individual, $\mathbf{f}_j = (f_{1,j}, \ldots, f_{C,j})$, where the weights of their effect on a gene g is $\mathbf{v}_g = (v_{g,1}, \ldots, v_{g,C})$ is modelled as:

$$P(y^{(2)}_{g,j} \,|\, \mathbf{f}_j, \mathbf{v}_g, \tau_g) = \mathcal{N}(y^{(2)}_{g,j} \,|\, \sum_{c=1}^{C} v_{g,c}\, f_{c,j}, \frac{1}{\tau_g}) \quad (7)$$

$$P(v_{g,c} \,|\, \alpha_c) = \mathcal{N}(v_{g,c} \,|\, 0, \frac{1}{\alpha_c}) \quad (8)$$

$$P(\alpha_c) = \Gamma(\alpha_c \,|\, a_\alpha, b_\alpha). \quad (9)$$

The gamma prior on the inverse covariances for each factor introduces automatic relevance detection (ARD) [5,6], driving the weights of unused factors to 0 and thereby switching them off. This is explained in more detail below.

3) Hidden factor model. Analogously to known factors, expression variability is modelled by linear effects from K hidden factors $\mathbf{X} = \{\mathbf{x}_1, \ldots, \mathbf{x}_K\}$:

$$P(y^{(3)}_{g,j} \,|\, \mathbf{x}_j, \mathbf{w}_g, \tau_g) = \mathcal{N}(y^{(3)}_{g,j} \,|\, \sum_{k=1}^{K} w_{g,k}\, x_{k,j}, \frac{1}{\tau_g}) \quad (10)$$

$$P(w_k, \beta_k) = \prod_{g=1}^{G} \mathcal{N}(w_{g,k} \,|\, 0, \frac{1}{\beta_k}) \quad (11)$$

$$P(x_{k,j}) = \mathcal{N}(x_{k,j} \,|\, 0, 1) \quad (12)$$

$$P(\beta_k) = \Gamma(\beta_k \,|\, a_\beta, b_\beta). \quad (13)$$

The factor activations $\mathbf{X}$ are random variables that are not observed, but instead inferred from the expression levels. Again, the ARD prior allows unused factors to be switched off. This forces the model to learn factors which have a broad effect on many expression levels. In experiments we used values $a_\alpha = 10^{-7}G$ and $b_\alpha = 10^{-1}G$, where G is the total number of gene probes. Similar prior settings were used for the weights of the known factors $\mathbf{v}_c$. We put a standard normal prior on the hidden factors, $x_{k,j} \sim \mathcal{N}(x_{k,j} \,|\, 0, 1)$.

Variational inference

As outlined in Methods we use variational Bayesian inference [7] for parameter learning in the framework. The basic principle of variational methods is to approximate the exact joint posterior distribution over all parameters by a factorised Q-distribution. Individual factors of the Q-distribution are refined by minimisation of the KL-divergence between the exact and the approximate distributions with respect to the parameters of a single factor. This leads to an iterative algorithm, updating individual factors of the approximate distribution given the state of all others. Here, we give the factorisations and update rules for the general framework and the individual models.

PEER framework. We approximate the exact joint posterior distribution over all parameters

$$P(\{\mathbf{Y}^{(m)}\}_{m=1}^{M}, \{\boldsymbol{\theta}^{(m)}\}_{m=1}^{M}, \,|\, \mathcal{D}) \quad (14)$$

by a factorised approximation over parameters for individual models

$$Q(\boldsymbol{\Theta}) = \prod_{m=1}^{M} Q(\boldsymbol{\theta}^{(m)}) Q(\mathbf{Y}^{(m)}). \quad (15)$$

Here we defined the abbreviation $\mathcal{D} = \{\mathbf{Y}, \{\mathcal{D}^{(m)}\}_{m=1}^{M}\}$, summarising all observed data; expression levels $\mathbf{Y}$ as well as model-specific data $\{\mathcal{D}^{(m)}\}_{m=1}^{M}$. Note that as the expression contributions $\mathbf{Y}^{(m)}$ are not observed they also resemble parameters that need to be inferred. Strictly speaking these are not treated as random variables of the model, but Gaussian messages that comprise the first and second moments of the expression variability contribution of a respective model. The distributions of parameters $\boldsymbol{\theta}^{(m)}$ for individual models are in turn factorised. The set $\boldsymbol{\Theta} = \{\boldsymbol{\theta}^{(1)}, \ldots, \boldsymbol{\theta}^{(M)}\}$ denotes the set of all parameters from all models.

The approximate Q-distributions are updated iteratively, taking the current state of all others into account. Update equations for a particular Q_i can be derived by functional minimisation of the KL-divergence between P and Q with respect to Q_i which leads to

$$\tilde{Q}(\Theta_i) \propto \exp\{\langle \log P(\mathcal{D}, \boldsymbol{\Theta})\rangle_{Q(\Theta_j), i \neq j}\}. \tag{16}$$

The term in the exponent is the expectation of the model log-likelihood under all other Q-distributions. Together with the expression data likelihood

$$P(\mathbf{Y} \mid \boldsymbol{\Theta}) = \mathcal{N}(\mathbf{Y} \mid \mathbf{Y}^{(1)} + \cdots + \mathbf{Y}^{(M)}, \boldsymbol{\Sigma}) \prod_{m=1}^{M} P(\mathbf{Y}^{(m)} \mid \boldsymbol{\theta}^{(m)}, \mathcal{D}^{(m)}) \tag{17}$$

this allows generic update rules for all model parameters to be derived. Substituting in Equation (15) for each $Q(\cdot)$, we obtain the following approximate distributions:

(Approximate distributions)

$$Q(\boldsymbol{\tau}) = \prod_{g=1}^{G} \Gamma(\tau_g \mid \tilde{a}_{\tau_g}, \tilde{b}_{\tau_g}) \tag{18}$$

$$Q(\mathbf{Y}^{(m)}) = \prod_{g=1}^{G} \prod_{j=1}^{J} \mathcal{N}(y_{g,j}^{(m)} \mid \tilde{m}_{Y_{g,j}^{(m)}}, \frac{1}{\tilde{\tau}_{Y_{g,j}^{(m)}}}), \tag{19}$$

and similar factorisations for each of the models (given below). The parameter update equations for the framework parameters follow as:

(Update rules)

$$\tilde{a}_{\tau_g} = a_\tau + \frac{1}{2} \sum_{j=1}^{J} \left\langle \left(y_{g,j} - \sum_{m=1}^{M} y_{g,j}^{(m)} \right)^2 \right\rangle \tag{20}$$

$$\tilde{b}_{\tau_g} = b_\tau + \frac{J}{2}. \tag{21}$$

Genotype effect model The update equations for the models introduced in the main text (Inference) follow similarly. For the models, we give the approximate factorisations employed, and the resulting update equations that are derived in identical manner to the treatment above.

(Approximate distributions)

$$Q(\mathbf{B}) = \prod_{n=1}^{N}\prod_{g=1}^{G} \text{Bernoulli}(b_{n,g}\,|\,\tilde{p}_{b_{n,g}}) \tag{22}$$

$$Q(\mathbf{U}) = \prod_{n=1}^{N}\prod_{g=1}^{G} \mathcal{N}(\mathbf{u}_{n,g}\,|\,\tilde{\mathbf{m}}_{\mathbf{u}_{n,g}}, \tilde{\mathbf{\Sigma}}_{\mathbf{u}_{n,g}}) \tag{23}$$

(Update rules)

$$\tilde{\Sigma}_{\mathbf{u}_{n,g}} = \mathbf{I} + \langle \tau_g \rangle \left\langle b_{n,g}^2 \right\rangle \sum_{j=1}^{J} \mathbf{s}_{n,j}^{\mathrm{T}} \mathbf{s}_{n,j} \tag{24}$$

$$\tilde{\mathbf{m}}_{\mathbf{u}_{n,g}} = \tilde{\Sigma}_{\mathbf{u}_{n,g}}^{-1} \left(\langle \tau_g \rangle \langle b_{n,g} \rangle \sum_{j=1}^{J} \mathbf{s}_{n,j} \left\langle z_{g,j}^{(1)\,\backslash n} \right\rangle \right) \tag{25}$$

$$\tilde{m}_{y_{g,j}^{(1)}} = \sum_{n=1}^{N} \langle b_{n,g} \rangle \langle \mathbf{u}_{n,g} \rangle \mathbf{s}_{n,j} \tag{26}$$

$$\tilde{\tau}_{y_{g,j}^{(1)}} = \left[\sum_{n=1}^{N} \left\langle b_{n,g}^2 \right\rangle \left\langle \mathbf{u}_{n,g}^2 \right\rangle \mathbf{s}_{n,j}^2 \right], \tag{27}$$

where we define

$$\left\langle z_{g,j}^{(1)\,\backslash n} \right\rangle = z_{g,j}^{(1)} - \sum_{m \neq n} \langle b_{m,g} \rangle \langle \mathbf{u}_{m,g} \rangle \mathbf{s}_{m,j} \tag{28}$$

and the residual expression dataset for the mth model

$$z_{g,j}^{(m)} = y_{g,j} - \sum_{l \neq m}^{M} y_{g,j}^{(l)}. \tag{29}$$

$$\tag{30}$$

The approximate posterior over the indicator variables can be obtained from

$$\tilde{p}_{b_{n,g}} \propto p_b \cdot \exp\left\{ -\frac{1}{2} \sum_{j=1}^{J} \left\langle \left(z_{g,j}^{(1)\,\backslash n} - b_{n,g} \boldsymbol{\theta}_{n,g} \mathbf{s}_{n,j} \right)^2 \right\rangle \right\}$$

$$(1 - \tilde{p}_{b_{n,g}}) \propto (1 - p_b) \cdot \exp\left\{ -\frac{1}{2} \sum_{j=1}^{J} \left\langle \left(z_{g,j}^{(1)\,\backslash n} \right)^2 \right\rangle \right\}, \tag{31}$$

which after normalisation gives rise to $\tilde{p}_{b_{n,g}}$.

$$\tag{32}$$

Known factor model is identical in treatment to the **hidden factor model**, without the need for updates of the factor activations. Thus, we only present the hidden factor model here.

$$ \tag{33} $$

(Approximate distributions)

$$Q(\mathbf{X}) = \prod_{j=1}^{J} \mathcal{N}(\mathbf{x}_j \,|\, \tilde{\mathbf{m}}_{\mathbf{x}_j}, \tilde{\Sigma}_{\mathbf{x}_j}) \tag{34}$$

$$Q(\mathbf{W}) = \prod_{g=1}^{G} \mathcal{N}(\mathbf{w}_g \,|\, \tilde{\mathbf{m}}_{\mathbf{w}_g}, \tilde{\Sigma}_{\mathbf{w}_g}) \tag{35}$$

$$Q(\boldsymbol{\beta}) = \prod_{k=1}^{K} \Gamma(\beta_k \,|\, \tilde{a}_{\beta_k}, \tilde{b}_{\beta_k}) \tag{36}$$

(Update rules)

$$\tilde{\Sigma}_{\mathbf{x}_j} = \Sigma_{\mathbf{x}_j} + \left\langle \mathbf{W}^T \mathrm{diag}\left(\boldsymbol{\tau}\right) \mathbf{W} \right\rangle \tag{37}$$

$$\tilde{\mathbf{m}}_{\mathbf{x}_j} = \tilde{\Sigma}_{\mathbf{x}_j}^{-1} \left\langle \mathbf{W}^{\mathrm{T}} \right\rangle \mathrm{diag}\left\langle \boldsymbol{\tau} \right\rangle \left(\left\langle \mathbf{z}_j^{(3)} \right\rangle \right) \tag{38}$$

$$\tilde{\Sigma}_{\mathbf{w}_g} = \mathrm{diag}\left\langle \boldsymbol{\beta} \right\rangle + \left\langle \tau_g \right\rangle \sum_{j=1}^{J} \left\langle \mathbf{x}_j \mathbf{x}_j^{\mathrm{T}} \right\rangle \tag{39}$$

$$\tilde{\mathbf{m}}_{\mathbf{w}_g} = \tilde{\Sigma}_{\mathbf{w}_g}^{-1} \left(\left\langle \tau_g \right\rangle \sum_{j=1}^{J} \left\langle \mathbf{x}_j \right\rangle \left(\left\langle \mathbf{z}_j^{(3)} \right\rangle \right) \right) \tag{40}$$

$$\tilde{m}_{y_{g,j}^{(3)}} = \sum_{k=1}^{K} \left\langle w_{g,k} \right\rangle \left\langle x_{j,k} \right\rangle \tag{41}$$

$$\tilde{\tau}_{y_{g,j}^{(3)}} = \left[\sum_{n=1}^{N} \left\langle b_{n,g}^2 \right\rangle \left\langle \mathbf{u}_{n,g}^2 \right\rangle \mathbf{s}_{n,j}^2 \right] \tag{42}$$

$$ \tag{43} $$

Initialisation. The initial states of hidden factor model weights $Q(\mathbf{w}_g)$ and levels $Q(\mathbf{x}_j)$ are determined from a PCA solution, and the weights for known factors $Q(\mathbf{v}_g)$ are initialised to the maximum likelihood estimate. The parameters for remaining Q distributions for all models are deterministically initialised to corresponding prior means. A random initialisation is possible as well, however, additional computation time is required for multiple restarts, and the inference becomes non-deterministic. We have not explored the implications of this alternative here as the maximum likelihood initialisation performs robustly well in practise.

Bottleneck approximation. The genetic association model accounts for additive association signals from all considered SNPs. The corresponding variational updates of the indicator variables in Equation (31) can be unstable in practise. In particular, if multiple correlated SNPs are in association to a single gene, variational learning is prone to being trapped in local optima, attributing the effect to only one of them. Hence, the inferred state of the indicator variables $\mathbf{B}$ depends on the order in which these updates are carried out. To obtain meaningful results, the update sequence needs to be randomised

and typically large numbers of restarts are required. This procedure implies prohibitive computational cost, particularly for large datasets. To avoid this additional computation, these updates are instead implemented greedily. For each gene g only a single non-zero entry in the indicator matrix is permitted, corresponding to the SNP with the greatest evidence for an association. This leads to a sparse association matrix $\mathbf{B}$.

VBQTL

Both the iterative (iVBQTL) and the fast variant (fVBQTL) of the studied algorithms use these update equations presented above. iVBQTL uses the full variational approximation with a specific update order of the $Q(\boldsymbol{\theta}_i)$ distributions (Algorithm 1b). In experiments, we used 3 iterations of the full model. Within each full iteration, the genetic model was iterated 3, known factor model 30 and hidden factor model 30 times.

To compare the eQTL detection performance of VBQTL with standard methods and previous studies, we do not directly evaluate the linkage probabilities $P(b_{n,g})$ which are obtained during learning. Instead, we apply the standard association model (Section Standard expression QTL model) on the residuals of the known and unknown factor models after convergence similarly to the traditional methods.

fVBQTL is a faster approximate variant of iVBQTL (Algorithm 1a). Rather than performing full inference in the model, the genetic part of the model is ignored when inferring the parameters for the factor models, which can be cast as a specific update schedule.

Algorithm 1 a fVBQTL

initialise Q distributions

for i iterations **do**
  (known factors)
  update $Q(\mathbf{v}_g), Q(\alpha_c)$
  (noise precision)
  update $Q(\tau_g)$
  (hidden factors)
  update $Q(\mathbf{w}_g), Q(\beta_k), Q(\mathbf{x}_j)$
  (noise precision)
  update $Q(\tau_g)$
end for
(genetic model)
update: $Q(u_{g,n}), Q(b_{n,g})$
(Out: Approximate posterior distribution of model parameters)

Algorithm 1b iVBQTL

initialise Q distributions

for i iterations **do**
  (genetic model)
  update: $Q(u_{g,n}), Q(b_{n,g})$
  (noise precision)
  update: $Q(\tau_g)$
  (known factors)
  update: $Q(\mathbf{v}_g), Q(\alpha_c)$
  (noise precision)
  update: $Q(\tau_g)$
  (hidden factors)
  update: $Q(\mathbf{w}_g), Q(\beta_k), Q(\mathbf{x}_j)$
  (noise precision)
  update: $Q(\tau_g)$
end for
(Out: Approximate posterior distribution of model parameters)

Simulation dataset

We simulated 80 diploid individuals with 100 SNPs and 400 probe expression measurements. The simulated minor allele frequency was 0.4 for each SNP, and the allele configuration $s_{n,j}$ of SNP n was encoded as $(1,0)$, $(1,1)$, or $(1,2)$, including a column for the mean. We independently simulated effects of known and hidden factors, as well as genetic associations, noise, and downstream effects. Noise level ψ_g of probe g was drawn from a normal distribution with mean 0 and inverse variance τ_g drawn from $\Gamma(3,1)$, $\psi_g \sim \mathcal{N}(0, \tau_g^{-1})$. We simulated associations between SNP genotypes and gene expression levels for 1% of

the SNP-gene pairs. The genetic weight $\theta_{g,n}$ for an association between probe g and SNP n was drawn from $\mathcal{N}(0,4)$. A total of 10 global factors affecting all gene expression levels were simulated. Individual factor levels $x_{j,k}$ for factor k were drawn from $\mathcal{N}(0,0.6)$. Weights $w_{k,g}$ of factor k for probe g were drawn from $N(0,\sigma_k^2)$, where $\sigma_k^2 \sim 0.8(\Gamma(2.5,0.6))^2$ for a heavy-tailed weight distribution. Three of the 10 simulated global factors were designated as known covariates $f_{c,j}$. Further three probes that had a simulated SNP association were designated to have downstream effects on 30 other probes. The effect of probe g on probe h in individual j was simulated as additive factor of $w'_{g,h} y_{g,j}$, where $w'_{g,h} \sim \mathcal{N}(8,0.8)$ for strong downstream effects, and $y_{g,j}$ is the expression level of probe g in individual j.

References

1. Lander E, Botstein D (1989) Mapping Mendelian Factors Underlying Quantitative Traits Using RFLP Linkage Maps. Genetics 121: 185–199.

2. Churchill GA, Doerge RW (1994) Empirical threshold values for quantitative trait mapping. Genetics 138: 963–971.

3. Stranger BEE, Nica ACC, Forrest MSS, Dimas A, Bird CPP, et al. (2007) Population genomics of human gene expression. Nature genetics 39: 1217.

4. Leek JT, Storey JD (2007) Capturing heterogeneity in gene expression studies by surrogate variable analysis. PLoS Genetics 3: e161.

5. Mackay DJC (1995) Probable networks and plausible predictions - a review of practical Bayesian methods for supervised neural networks. Network: Computation in Neural Systems 6: 469–505.

6. Neal RM (1996) Bayesian Learning for Neural Networks. Springer.

7. Jordan M, Ghahramani Z, Jaakkola T, Saul L (1999) An introduction to variational methods for graphical models. Machine Learning 37: 183-233.
