# Supplementary material for: A Bayesian Framework to Account for Complex Non-Genetic Factors in Gene Expression Levels Greatly Increases Power in eQTL Studies
Source: PLoS Comput Biol. 2010 May 6;6(5):e1000770. doi: 10.1371/journal.pcbi.1000770 (PMC2865505; doi:10.1371/journal.pcbi.1000770)
Supplement: Text S2 — Supplementary results. (0.86 MB PDF) [file pcbi.1000770.s002.pdf]

Supplementary Results

Finding *trans* associations in human.

Associating 10,000 probes against 34,143 linkage regions using standard permutation testing techniques is computationally infeasible. However, SNPs that have the same number of each genotype in a population, can be mapped onto each other via a permutation. We call such SNPs permutation equivalent, since if we performed a large enough number of permutations, they would share the same significance cutoff value. This can be used to overcome the computational cost barrier. We grouped SNPs into permutation equivalence classes, for instance there are 1579 unique ones in the CEU population. We precalculated $10,000$ permutation LOD scores for each of the $10,000$ probes against a member of each equivalence class and used the 0.01% tail of these scores as a significance cutoff value. When testing for significance of a region we used the cutoff values for the equivalence classes of the SNPs in that region. As in the *cis*-study, an association was called when the LOD score for at least one SNP in a linkage region exceeded the LOD-score cutoff for all SNPs in that region. With this FPR the number of expected false associations is still immense. We thus added 5 to the LOD score cutoff to obtain a more stringent threshold.

We used the procedure outlined above to test SNPs in each of 34,143 linkage blocks as defined by recombination hotspots [1, 2] for association with the 10,000 most variable gene probes for the three HapMap populations. Since full permutation testing on the complete data is computationally infeasible, we restricted the tests to SNPs without missing values and used a conservative correction for the low number of permutations as described above. For the CEU population we found 294 standard eQTLs and 774 VBeQTLs with the same 30 factors learned as the *cis* study. This corresponds to 205 and 537 unique probes respectively.

To differentiate between *cis* and *trans* associations, we called an association *trans* if it is at least 5Mb away from the probe midpoint. Using this criterion, 84% of the standard eQTLs and 87% of the VBeQTLs were in *cis* (Figure 1). Counting only *trans* associations, we found 162 VBeQTLs and 126 standard eQTLs in at least one population, and 48 VBeQTLs and 32 standard eQTLs in at least two populations.

Standard<br>Population	CEU (47)	YRI (78)	CHB+JPT (46)
CEU (47)	47 (100%)	18 (38%)	22 (47%)
YRI (78)	18 (23%)	78 (100%)	18 (23%)
CHB+JPT (46)	22 (48%)	18 (39%)	46 (100%)
All populations	13		
> 1 populations	32		
Any population	126		

fVBQTL<br>Population	CEU (72)	YRI (87)	CHB+JPT (76)
CEU (72)	72 (100%)	26 (36%)	41 (57%)
YRI (87)	26 (30%)	87 (100%)	31 (36%)
CHB+JPT (76)	41 (54%)	31 (41%)	76 (100%)
All populations	25		
> 1 populations	48		
Any population	162		

Table 1: Count and percent overlap between probes in *trans* associations on different populations using standard method and after using fVBQTL.

The same analysis was carried out on the remaining two HapMap populations identifying unique associations in all populations and their overlap. Similarly to *cis* eQTLs, the additional *trans* associations replicated in different populations with rates between 30% (YRI in CEU) and 57% (CEU in CHB+JPT) (Table 1).

References

1. McVean GA, Myers SR, Hunt S, Deloukas P, Bentley DR, et al. (2004) The fine-scale structure of recombination rate variation in the human genome. Science 304: 581–584.

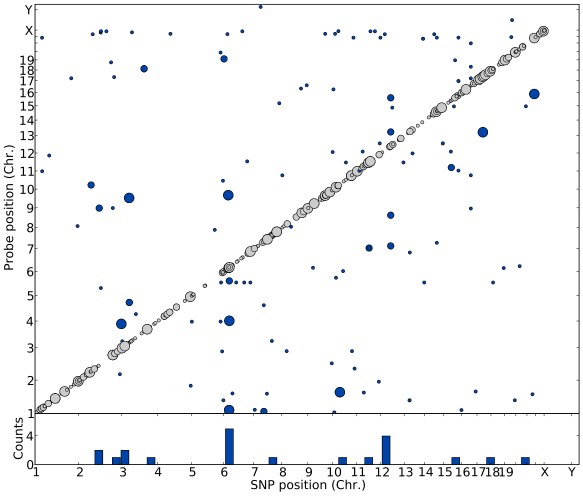

(a) Distribution of standard eQTLs

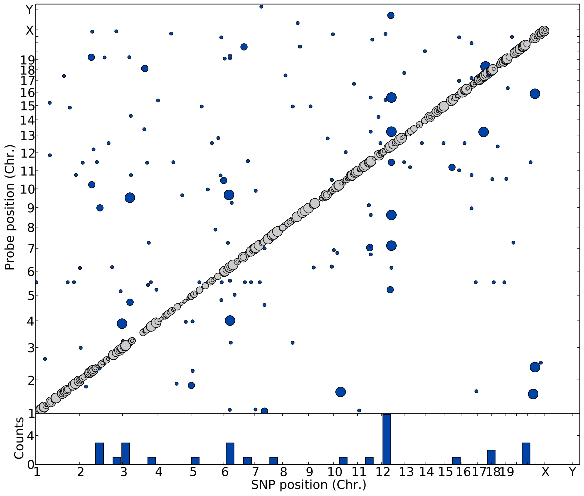

(b) Distribution of VBQTLs

Figure 1: Distribution of significant associations (FPR=10^{-8}) for **(a)** Standard eQTLs and **(b)** VBeQTLs pooled over the three HapMap populations. Grey: *cis* associations (SNP distance to probe midpoint $<$ 5Mb), blue: *trans* associations. The size of the dot indicates the overlap between populations (one, two or all three populations). 13 standard and 25 VBeQTLs are replicated in all populations; some of them overlap due to proximity of the linkage regions. The association counts below the diagrams sum up *trans* associations shared in at least two populations.

2. Winckler W, Myers SR, Richter DJ, Onofrio RC, McDonald GJ, et al. (2005) Comparison of fine-scale recombination rates in humans and chimpanzees. Science 308: 107–111.
