# Supplementary material for: A Bayesian Framework to Account for Complex Non-Genetic Factors in Gene Expression Levels Greatly Increases Power in eQTL Studies
Source: PLoS Comput Biol. 2010 May 6;6(5):e1000770. doi: 10.1371/journal.pcbi.1000770 (PMC2865505; doi:10.1371/journal.pcbi.1000770)
Supplement: Figure S2 — Sensitivity of recovering human eQTLs for alternative eQTL models. (a–b) Using a standard nested model for expression values, performing chi-squared tests with one degree of freedom on the log likelihood ratio for adding the genetic association term to the model. (c–d) Using a standard nested model for ranks of expression values, performing t tests with N-2 degrees of freedom as described in Supplementary Methods. Bonferroni correction to 1% false positive rate was used for both methods to correct for multiple testing as detailed in Text S1. (0.23 MB PDF) [file pcbi.1000770.s004.pdf]

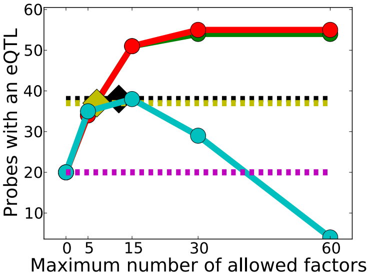

(a) Immediate (cis) eQTLs (t test)

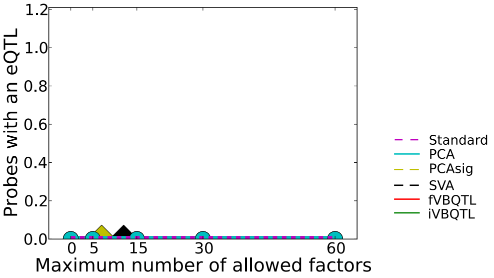

(b) Downstream (trans) eQTLs (t test)

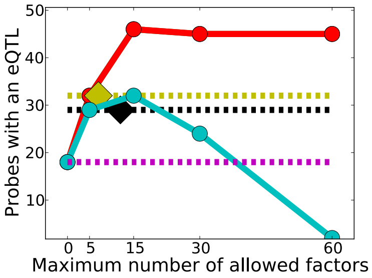

(c) Immediate (cis) eQTLs
(permutation test)

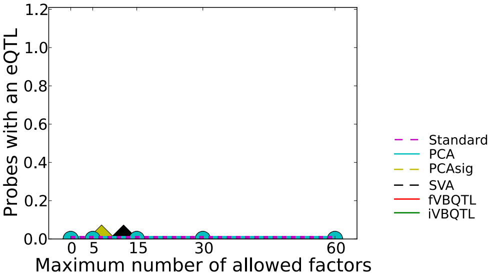

(d) Downstream (trans) eQTLs
(permutation test)
