# Supplementary material for: A Bayesian Framework to Account for Complex Non-Genetic Factors in Gene Expression Levels Greatly Increases Power in eQTL Studies
Source: PLoS Comput Biol. 2010 May 6;6(5):e1000770. doi: 10.1371/journal.pcbi.1000770 (PMC2865505; doi:10.1371/journal.pcbi.1000770)
Supplement: Figure S4 — Sensitivity of recovering mouse eQTLs for alternative eQTL models. (a–b) Using a standard model for expression values, performing 2-tailed t tests on the statistic based on correlation coefficient between expression level and genotype. (c–d) Similar test for ranks of expression values. Bonferroni correction to 0.1% false positive rate was used for both methods to correct for multiple testing as detailed in Text S1. (0.25 MB PDF) [file pcbi.1000770.s006.pdf]

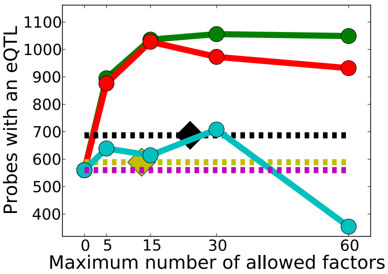

(a) Immediate (cis) eQTLs (t test)

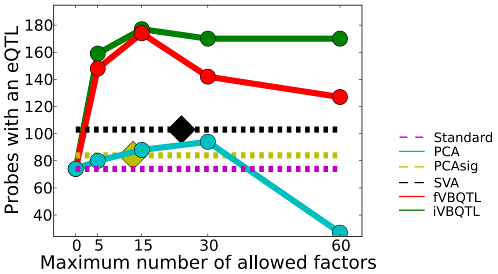

(b) Downstream (trans) eQTLs (t test)

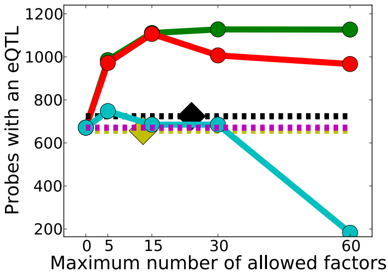

(c) Immediate (cis) eQTLs
(permutation test)

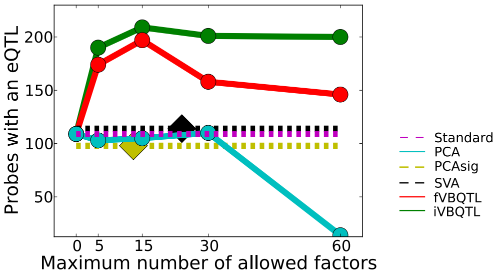

(d) Downstream (trans) eQTLs
(permutation test)
