# Supplementary material for: A Bayesian Framework to Account for Complex Non-Genetic Factors in Gene Expression Levels Greatly Increases Power in eQTL Studies
Source: PLoS Comput Biol. 2010 May 6;6(5):e1000770. doi: 10.1371/journal.pcbi.1000770 (PMC2865505; doi:10.1371/journal.pcbi.1000770)
Supplement: Table S1 — Number of probes with a cis association for individual chromosomes and per-probe false discovery rate for the considered populations (per-probe FPR = 0.100%, Bonferroni corrected for testing multiple SNPs per probe, 2-tailed t test) on raw expression data (Standard) and after accounting for hidden factors (fVBQTL). (0.02 MB PDF) [file pcbi.1000770.s007.pdf]

Chr.	1	2	3	4	5	6	7	8	9	10	11	12	13	14	15	16	17	18	19	20	21	22	X	Y	**Total**	**FDR**
Probes	1009	644	540	384	449	571	468	338	387	380	545	520	189	330	348	426	549	154	618	266	120	238	328	15	9816	-
**CEU**																										
Standard	23	21	12	24	14	26	18	12	3	17	21	24	5	16	15	21	35	9	29	8	8	14	7	0	382	2.57 %
fVBQTL	61	69	53	57	45	83	44	36	12	48	61	68	16	41	32	55	82	20	69	29	17	30	23	0	1051	0.93 %
**YRI**																										
Standard	37	32	23	19	21	42	27	17	9	27	31	30	9	24	16	24	38	12	30	18	8	26	9	0	529	1.86 %
fVBQTL	79	94	75	48	56	91	66	38	17	58	79	65	26	48	48	59	94	22	77	40	19	43	27	0	1269	0.77 %
**ASI**																										
Standard	36	37	19	28	19	48	30	15	9	24	33	36	10	19	12	24	43	16	42	16	10	19	9	0	554	1.77 %
fVBQTL	91	105	88	55	58	111	73	55	19	59	87	78	31	56	52	61	109	30	96	43	22	37	28	0	1444	0.68 %
**pooled**																										
Standard	68	77	56	48	42	79	52	32	14	46	48	66	21	39	34	43	82	21	71	31	19	37	19	0	1045	0.94 %
fVBQTL	159	191	158	115	120	202	138	101	36	120	168	159	54	104	96	113	181	51	170	78	33	85	60	4	2696	0.36 %
