# Supplementary material for: A Bayesian Framework to Account for Complex Non-Genetic Factors in Gene Expression Levels Greatly Increases Power in eQTL Studies
Source: PLoS Comput Biol. 2010 May 6;6(5):e1000770. doi: 10.1371/journal.pcbi.1000770 (PMC2865505; doi:10.1371/journal.pcbi.1000770)
Supplement: Table S2 — Magnitude and fraction of overlap between probes with a Standard of fVBQTL cis eQTL respectively, for different populations and methods. Total numbers for each population and method are given in parenthesis after the population. 955 probes had a standard eQTL in some population, and 148 in every population. 2236 probes had a fVBQTL eQTL in some population, and 477 in every population. (0.02 MB PDF) [file pcbi.1000770.s008.pdf]

		CEU (382)	YRI (529)	CHB+JPT (554)	Pooled (1045)
		**Standard eQTLs**			
Standard	CEU (382)	382 (100%)	194 (50%)	236 (61%)	356 (93%)
	YRI (529)	194 (36%)	529 (100%)	228 (43%)	409 (77%)
	CHB+JPT (554)	236 (42%)	228 (41%)	554 (100%)	490 (88%)
	Pooled (1045)	356 (34%)	409 (39%)	490 (46%)	1045 (100%)
fVBQTL	CEU (1051)	365 (34%)	282 (26%)	358 (34%)	662 (62%)
	YRI (1269)	276 (21%)	510 (40%)	356 (28%)	675 (53%)
	CHB+JPT (1444)	305 (21%)	322 (22%)	531 (36%)	788 (54%)
	Pooled (2696)	370 (13%)	486 (18%)	527 (19%)	1028 (38%)
		**fVBQTL eQTLs**			
		CEU (1051)	YRI (1269)	CHB+JPT (1444)	Pooled (2696)
Standard	CEU (382)	365 (95%)	276 (72%)	305 (79%)	370 (96%)
	YRI (529)	282 (53%)	510 (96%)	322 (60%)	486 (91%)
	CHB+JPT (554)	358 (64%)	356 (64%)	531 (95%)	527 (95%)
	Pooled (1045)	662 (63%)	675 (64%)	788 (75%)	1028 (98%)
fVBQTL	CEU (1051)	1051 (100%)	591 (56%)	717 (68%)	1007 (95%)
	YRI (1269)	591 (46%)	1269 (100%)	697 (54%)	1120 (88%)
	CHB+JPT (1444)	717 (49%)	697 (48%)	1444 (100%)	1350 (93%)
	Pooled (2696)	1007 (37%)	1120 (41%)	1350 (50%)	2696 (100%)
