# Supplementary material for: A Bayesian Framework to Account for Complex Non-Genetic Factors in Gene Expression Levels Greatly Increases Power in eQTL Studies
Source: PLoS Comput Biol. 2010 May 6;6(5):e1000770. doi: 10.1371/journal.pcbi.1000770 (PMC2865505; doi:10.1371/journal.pcbi.1000770)
Supplement: Table S3 — Overlap of VBQTLs in one population (2.) with standard eQTLs found when pooling the other two populations (3.). Overlaps are given both for all QTLs (2. & 3.) and only for additional ones (2. - 1. & 3. - 1.) compared to standard eQTLs in the population. Per-probe eQTL FPR = 0.1%, Bonferroni corrected for testing multiple SNPs per probe, 2-tailed t test. (0.01 MB PDF) [file pcbi.1000770.s009.pdf]

Population	1. eQTLs	2. fVBQTLs	3. Pooled eQTLs	2. & 3.	2. - 1.	3. - 1.	(2. - 1.) &(3. - 1.)
CEU	382	1051	871	485	686	582	204
YRI	529	1269	796	476	759	507	188
CHB+JPT	554	1444	709	501	913	378	170
