# Supplementary material for: A Bayesian Framework to Account for Complex Non-Genetic Factors in Gene Expression Levels Greatly Increases Power in eQTL Studies
Source: PLoS Comput Biol. 2010 May 6;6(5):e1000770. doi: 10.1371/journal.pcbi.1000770 (PMC2865505; doi:10.1371/journal.pcbi.1000770)
Supplement: Table S4 — Pearson correlation coefficient between top 6 factors learned on the pooled HapMap data, and 4 indicator variables relating to the background of the individual. Correlations with absolute value above 0.6 are highlighted. (0.01 MB PDF) [file pcbi.1000770.s010.pdf]

| Factor | 1 | 2 | 3 | 4 | 5 | 6 |
|---|---|---|---|---|---|---|
| Gender | 0.12 | 0.16 | **-0.81** | 0.19 | 0.08 | -0.00 |
| CEU | **0.68** | -0.47 | -0.21 | -0.04 | -0.27 | 0.04 |
| CHB+JPT | -0.43 | 0.28 | -0.24 | **-0.64** | -0.08 | 0.03 |
| YRI | -0.25 | 0.19 | 0.46 | **0.69** | 0.35 | -0.08 |
