# Supplementary material for: A Bayesian Framework to Account for Complex Non-Genetic Factors in Gene Expression Levels Greatly Increases Power in eQTL Studies
Source: PLoS Comput Biol. 2010 May 6;6(5):e1000770. doi: 10.1371/journal.pcbi.1000770 (PMC2865505; doi:10.1371/journal.pcbi.1000770)
Supplement: Table S5 — Summary statistics for method performances on the human chromosome 19 dataset presented in the main text. The parameters for different methods are varied by the number of allowed factors K (PCA, VBQTL) or by the significance cutoff α (PCAsig, SVA). Hidden factor summary is given by the number of factors found and the variance explained by the hidden factor effects. The number of probes with a cis and trans eQTL, as well as the sensitivity and specificity of recovering probes with a standard eQTL are given. Per-probe eQTL FPR = 0.001, Bonferroni corrected for testing multiple SNPs per probe, 2-tailed t test. (0.02 MB PDF) [file pcbi.1000770.s011.pdf]

Method	K	α	Factors found	Variance explained	*cis* probes	*cis* spec.	*cis* sens.	*trans* probes	*trans* spec.	*trans* sens.
Standard	–	–	0	0.00	20	1.00	1.00	0	0.00	0.00
PCA	5	–	5	0.52	35	0.54	0.95	0	0.00	0.00
PCA	15	–	15	0.70	38	0.45	0.85	0	0.00	0.00
PCA	30	–	30	0.82	29	0.45	0.65	0	0.00	0.00
PCA	60	–	60	0.94	4	0.75	0.15	0	0.00	0.00
PCAsig	–	0.01	7	0.56	37	0.51	0.95	0	0.00	0.00
PCAsig	–	0.1	7	0.56	37	0.51	0.95	0	0.00	0.00
PCAsig	–	0.3	7	0.56	37	0.51	0.95	0	0.00	0.00
SVA	–	0.01	12	0.65	38	0.50	0.95	0	0.00	0.00
SVA	–	0.1	12	0.65	38	0.50	0.95	0	0.00	0.00
SVA	–	0.3	12	0.65	38	0.50	0.95	0	0.00	0.00
fVBQTL	5	–	5	0.52	34	0.59	1.00	0	0.00	0.00
fVBQTL	15	–	15	0.69	51	0.39	1.00	0	0.00	0.00
fVBQTL	30	–	30	0.70	55	0.36	1.00	0	0.00	0.00
fVBQTL	60	–	60	0.70	55	0.36	1.00	0	0.00	0.00
iVBQTL	5	–	5	0.52	34	0.59	1.00	0	0.00	0.00
iVBQTL	15	–	15	0.69	51	0.39	1.00	0	0.00	0.00
iVBQTL	30	–	30	0.70	54	0.37	1.00	0	0.00	0.00
iVBQTL	60	–	60	0.70	54	0.37	1.00	0	0.00	0.00
