# Supplementary material for: A Bayesian Framework to Account for Complex Non-Genetic Factors in Gene Expression Levels Greatly Increases Power in eQTL Studies
Source: PLoS Comput Biol. 2010 May 6;6(5):e1000770. doi: 10.1371/journal.pcbi.1000770 (PMC2865505; doi:10.1371/journal.pcbi.1000770)
Supplement: Table S6 — Summary statistics for method performances on the yeast dataset presented in the main text. The parameters for different methods are varied by the number of allowed factors K (PCA, VBQTL) or by the significance cutoff α (PCAsig, SVA). Hidden factor summary is given by the number of factors found and the variance explained by the hidden factor effects. The number of probes with a cis and trans eQTL, as well as the sensitivity and specificity of recovering probes with a standard eQTL are given. Per-probe eQTL FPR = 0.001, Bonferroni corrected for testing multiple SNPs per probe, 2-tailed t test. (0.02 MB PDF) [file pcbi.1000770.s012.pdf]

Method	K	α	Factors found	Variance explained	*cis* probes	*cis* spec.	*cis* sens.	*trans* probes	*trans* spec.	*trans* sens.
Standard	–	–	0	0.00	445	1.00	1.00	746	1.00	1.00
PCA	5	–	5	0.28	478	0.77	0.82	501	0.79	0.53
PCA	15	–	15	0.53	481	0.64	0.69	132	0.77	0.14
PCA	30	–	30	0.70	392	0.60	0.53	57	0.75	0.06
PCA	60	–	60	0.86	105	0.66	0.16	5	1.00	0.01
PCAsig	–	0.01	7	0.34	468	0.72	0.76	229	0.80	0.25
PCAsig	–	0.1	7	0.34	468	0.72	0.76	229	0.80	0.25
PCAsig	–	0.3	7	0.34	468	0.72	0.76	229	0.80	0.25
SVA	–	0.01	14	0.52	482	0.65	0.71	144	0.78	0.15
SVA	–	0.1	14	0.52	482	0.65	0.71	144	0.78	0.15
SVA	–	0.3	14	0.52	482	0.65	0.71	144	0.78	0.15
fVBQTL	5	–	5	0.34	547	0.72	0.89	409	0.81	0.45
fVBQTL	15	–	15	0.55	668	0.59	0.88	364	0.80	0.39
fVBQTL	30	–	30	0.62	719	0.54	0.87	349	0.79	0.37
fVBQTL	60	–	60	0.62	722	0.54	0.87	348	0.78	0.37
iVBQTL	5	–	5	0.32	616	0.68	0.95	650	0.76	0.66
iVBQTL	15	–	15	0.50	785	0.54	0.96	694	0.73	0.68
iVBQTL	30	–	30	0.57	821	0.52	0.95	746	0.71	0.71
iVBQTL	60	–	60	0.57	825	0.51	0.95	739	0.71	0.70
