# Supplementary material for: A Bayesian Framework to Account for Complex Non-Genetic Factors in Gene Expression Levels Greatly Increases Power in eQTL Studies
Source: PLoS Comput Biol. 2010 May 6;6(5):e1000770. doi: 10.1371/journal.pcbi.1000770 (PMC2865505; doi:10.1371/journal.pcbi.1000770)
Supplement: Table S7 — Summary statistics for method performances on the mouse dataset presented in the main text. The parameters for different methods are varied by the number of allowed factors K (PCA, VBQTL) or by the significance cutoff α (PCAsig, SVA). Hidden factor summary is given by the number of factors found and the variance explained by the hidden factor effects. The number of probes with a cis and trans eQTL, as well as the sensitivity and specificity of recovering probes with a standard eQTL are given. Per-probe eQTL FPR = 0.001, Bonferroni corrected for testing multiple SNPs per probe, 2-tailed t test. (0.02 MB PDF) [file pcbi.1000770.s013.pdf]

Method	K	α	Factors found	Variance explained	*cis* probes	*cis* spec.	*cis* sens.	*trans* probes	*trans* spec.	*trans* sens.
Standard	–	–	0	0.00	560	1.00	1.00	369	1.00	1.00
PCA	5	–	5	0.25	639	0.84	0.96	418	0.76	0.86
PCA	15	–	15	0.48	614	0.82	0.90	409	0.72	0.80
PCA	30	–	30	0.74	708	0.70	0.88	488	0.59	0.78
PCA	60	–	60	0.91	354	0.82	0.52	178	0.76	0.37
PCAsig	–	0.01	12	0.39	601	0.84	0.91	376	0.76	0.77
PCAsig	–	0.1	13	0.41	589	0.85	0.90	371	0.75	0.76
PCAsig	–	0.3	13	0.41	589	0.85	0.90	371	0.75	0.76
SVA	–	0.01	24	0.67	687	0.74	0.91	501	0.58	0.79
SVA	–	0.1	24	0.67	687	0.74	0.91	501	0.58	0.79
SVA	–	0.3	24	0.67	687	0.74	0.91	501	0.58	0.79
fVBQTL	5	–	5	0.32	876	0.63	0.98	590	0.56	0.90
fVBQTL	15	–	15	0.51	1028	0.54	0.99	716	0.46	0.89
fVBQTL	30	–	30	0.67	973	0.56	0.98	657	0.49	0.88
fVBQTL	60	–	60	0.70	932	0.59	0.98	626	0.51	0.87
iVBQTL	5	–	5	0.32	895	0.62	0.99	613	0.55	0.91
iVBQTL	15	–	15	0.51	1036	0.53	0.99	723	0.46	0.90
iVBQTL	30	–	30	0.55	1056	0.52	0.99	729	0.46	0.90
iVBQTL	60	–	60	0.55	1049	0.53	0.99	728	0.45	0.90
